# 2334 Neural correlates of externally Versus internally guided dance-based therapies for people with Parkinson’s disease

**DOI:** 10.1017/cts.2018.99

**Published:** 2018-11-21

**Authors:** Amrit Kashyap, Madeleine Hackney, Venkatagiri Krishnamurthy, Lisa Krishnamurthy, Krish Sathian, Bruce Crosson, Steve Wolf, Daniel Corcos, Jonathan Drucker, Marian Evatt, Gopi Kaundinya, Aaron Bozzorg, Ariel Hart

**Affiliations:** 1 Department of Radiology & Imaging Sciences, Emory University; 2 Department of Neurology, Emory University

## Abstract

OBJECTIVES/SPECIFIC AIMS: Parkinson’s disease (PD) is a condition that affects over a million Americans, and despite current medical therapies, the progression of the disease results in impaired generation of internally timed or guided (IG) movements. To address this loss of motor function, previous rehabilitation therapies have focused on remediating the affected striatal-thalamic-cortical circuits (STC), primarily thought to be responsible in generating timed motor patterns. However, given the disease leads to the cell death of dopaminergic cells that are essential for proper STC function, we propose a motor therapy aimed at utilizing a compensatory parallel cerebellar-thalamic-cortical (CTC) pathway, recruited to perform externally guided (EG) movements, in which gait initiation is driven from sensory input. Our previous study has shown efficacy in our novel argentine tango therapy and improves behavioral measures above the relevant MCID threshold, but it has not been established that the CTC are in the causal pathway that are responsible for these changes. Using neural measures from task fMRI, we have begun to characterize networks that have changed and quantify any associations with behavioral metrics. METHODS/STUDY POPULATION: Patients were randomly assigned to an IG (n=18), EG (n=18), or education contact control (n=14). Participants were assessed preintervention and postintervention for behavioral motor and cognitive measures and neurophysiologically with task based fMRI. In the task, participants performed a foot tapping task under both IG (tap their foot in previously learned rhythm) or EG (tap immediately after receiving a tactile cue on their hand) conditions. The fMRI data were preprocessed using AFNI and registered to MNI standard space. The brainnetome atlas was applied and the average time series of each region of interest (ROI) was used to increase the signal to noise ratio. The activation of these ROI with respect to the stimulus was modeled using GLM, and we estimated the area under the curve during the task blocks. A 1-way ANOVA analysis on these betas were performed between the pre and the post intervention time points and the ROIs that were above a significance of 0.95 were identified and corrected for multiple comparisons. The change in beta in all ROIs for each individual were calculated and then correlated with the changes in the behavioral data, to see which changes in ROI areas matched the best with the behavioral changes. RESULTS/ANTICIPATED RESULTS: The EG group showed significant changes only in the EG task in 2 areas—inferior frontal gyrus and inferior temporal sulcus. Correlating to the cognitive behavioral measures show reduced error from the Inferior frontal gyrus (corr>0.5) best reflect changes in observed. There were no changes to either the STC or the CTC pathways. The IG group showed no changes behaviorally and showed no changes neurally as well. The control group showed no changes behaviorally, but neuronally certain DMN nodes, such as the precuneus and inferior temporal regions showed a significant change for both tasks. DISCUSSION/SIGNIFICANCE OF IMPACT: Addressing the damaged STC pathway directly through IG therapy may not be effective. The EG therapy may not be able to enhance the STC pathway. However, the therapy appears to utilize new areas in the frontal regions and correlates with positively with changes in spatial memory and balance tasks. Contrary to our hypothesis the CTC circuit was not upregulated for performance of the IG or EG task, but therapy may have enhanced recruitment of other cognitively engaged areas. The educational control group interestingly showed changes in the DMN network, which has been shown to be linked to attention during tasks blocks.

